# Arthroscopic Inlay Biceps Tenodesis Using a Tendon-Docking Anchor

**DOI:** 10.1016/j.eats.2024.103284

**Published:** 2024-10-28

**Authors:** Mark G. Soliman, Katherine S. Worcester, Thomas E. Herron, Kevin F. Bonner

**Affiliations:** a Eastern Virginia Medical School, Old Dominion University, Norfolk, Virginia, U.S.A.; b Jordan-Young Institute for Orthopaedic Surgery and Sports Medicine, Virginia Beach, Virginia, U.S.A.; c Orthopaedic Research of Virginia, Henrico, Virginia, U.S.A.

## Abstract

Pathology of the long head of the biceps tendon can be treated surgically with a multitude of tenodesis techniques; however, there is a lack of consensus on which technique provides the most optimal outcomes. Commonly used methods include inlay tenodesis with a bone tunnel and interference screw construct and onlay tenodesis with anchors or unicortical buttons. Although current methods typically provide satisfactory outcomes, many surgeons believe complications and failure rates remain suboptimal across techniques. In this article, we present an arthroscopic suprapectoral biceps tenodesis technique using an anchor device that was developed to address the shortcomings of current techniques, optimize outcomes, and minimize risk.

Long head of the biceps tendon (LHBT) pathology is common and can occur in isolation or concomitantly with other shoulder pathologies, including rotator cuff disease and superior labral tearing. It can be a significant source of shoulder pain and decreased function and is often addressed surgically with tenodesis or tenotomy when conservative treatment fails. Biceps tenodesis (BT) has advantages over tenotomy, including maintenance of the muscle length-tension relationship and decreased complications such as spasm, cramping, and cosmetic deformity (Popeye sign).^1^^,^^2^ Although many tenodesis techniques have been developed to treat LHBT pathology, there is no clear consensus on which technique provides superior outcomes.^2^^,^^3^

BT can be performed arthroscopically or in an open manner, with many variations in approach, fixation, and location. Two of the most common and debated approaches are inlay BT, in which the LHBT is secured within a bone socket by means of an interference screw (IS), expansion device, bicortical button, or implant-free suture fixation, and onlay BT, in which the LHBT is secured to the cortical bone surface typically with anchors or unicortical buttons.^3^^,^^4^ The location of the BT within the suprapectoral versus subpectoral region, as well as arthroscopic versus open, has been substantially studied and is also a point of controversy.^3^, ^4^, ^5^ Historically, most tenodeses were performed in the suprapectoral location, either in an open manner or arthroscopically. Approximately a decade ago, there was a shift toward performing tenodesis in the subpectoral region because it was believed this would alleviate symptomatic bicipital groove pain, as well as optimize restoration of the length and tension of the LHBT.^6^ Recent meta-analyses and systematic reviews, however, have not shown significant differences in outcomes between tenodesis locations, including the incidence of postoperative bicipital groove pain.^3^^,^^5^^,^^7^, ^8^, ^9^ Over the past 5 to 10 years, there has been a trend toward onlay fixation as opposed to a tunnel and IS construct because of reports of a higher incidence of delayed Popeye deformity that was the result of iatrogenic tendon damage during screw insertion.^10^ Furthermore, bone tunnels can create larger stress risers, and have been associated with postoperative fractures after subpectoral tenodesis.^11^^,^^12^ However, more recent literature has suggested that tenodesis with onlay fixation may substantially elongate or even fail and not heal where the tendon is fixed at time zero.^13^^,^^14^ As a result of these shortcomings and the evolution of outcome data, variation in opinion and tenodesis technique remains, with no clear consensus.

In this article, we describe an arthroscopic suprapectoral BT technique that uses a PEEK (polyether ether ketone) anchor that was developed to address the shortcomings of current methods, optimize outcomes, and minimize risk. This technique was developed with the understanding that it must be easy to obtain proficiency, it must be reproducible and safe, and it must yield reliable results. The TIGHT-N Anchor (DePuy Synthes, Raynham, MA) is a BT device with a unique half-sheath design that protects the tendon from iatrogenic damage while incorporating the potential benefits of healing within a bone tunnel. The flanges of the device compress and then expand once seated in a subcortical manner, which has been shown to yield extremely high and consistent pullout strength.^15^

## Surgical Technique

The described arthroscopic technique is outlined in Video 1. Pearls and pitfalls are presented in Table 1. This procedure can be performed with the patient in either the beach-chair or lateral decubitus position. We prefer beach-chair positioning with the operative extremity in an arm holder or the use of an assistant to position the arm. A standard posterior viewing portal and anterior working portal are established to perform a diagnostic arthroscopy with a 30° arthroscope. If indicated for tenodesis, the biceps tendon is released from its attachment using a radiofrequency ablation device.

**Table 1:** Pearls and Pitfalls of TIGHT-N Biceps Tenodesis Technique

Pearls	Pitfalls
In terms of implant sizing, in our experience, the 7-mm (medium) implant is used in 65%-70% of cases, and the 5.5-mm implant is used in the remainder. If in doubt, the surgeon should err on the side of using the larger, 7-mm size. An 8.5-mm implant is almost never used.	Regarding the fixation location, the implant is not intended for subpectoral biceps tenodesis.
For accessory biceps portal placement, the triangle method and a spinal needle should be used to determine optimal placement of the accessory biceps portal for tendon exteriorization and reaming.	In terms of finding the biceps, in the subacromial space, it is easier to find the biceps tendon and groove more distally toward the pectoralis tendon. There is almost always a small vertically oriented vessel just lateral to the groove.
In terms of exteriorization of the tendon, forward flexion of the shoulder and elbow can aid with exteriorizing sufficient tendon length. Clamping the distal tendon at the skin can assist with tendon management when suturing and docking the tendon to the anchor implant.	To avoid obstruction in the deltoid muscle, a malleable sled retractor can be introduced through the accessory biceps portal and used as an alternative to a cannula during reaming and delivery of the implant through the deltoid to the aperture of the tunnel.
Regarding workflow, tenodesis should be performed prior to rotator cuff repair to minimize extravasation of the deltoid.	

After the intra-articular portion of the procedure, the arthroscope is moved to the subacromial space, and a working lateral portal is made. A thorough bursectomy is performed to obtain optimal visualization of the rotator cuff and bicipital groove anterior. While viewing with a 70° scope from the posterior portal into the anterior subacromial space (aiming the scope distally), the shoulder and elbow are forward flexed. The shoulder is typically externally rotated 15° to 25° to bring the bicipital groove lateral, which can make visualization more optimal. There is typically a vessel that runs just parallel to the bicipital groove on the lateral side that can assist in identifying the groove. The bicipital groove is much easier to identify more distally in the anterior subacromial space. Attempting to isolate the groove proximally is often more challenging. The tendon can be palpated distally within the groove, and this is where opening and decompressing the groove in a proximal direction are initiated.

**Fig 1:**
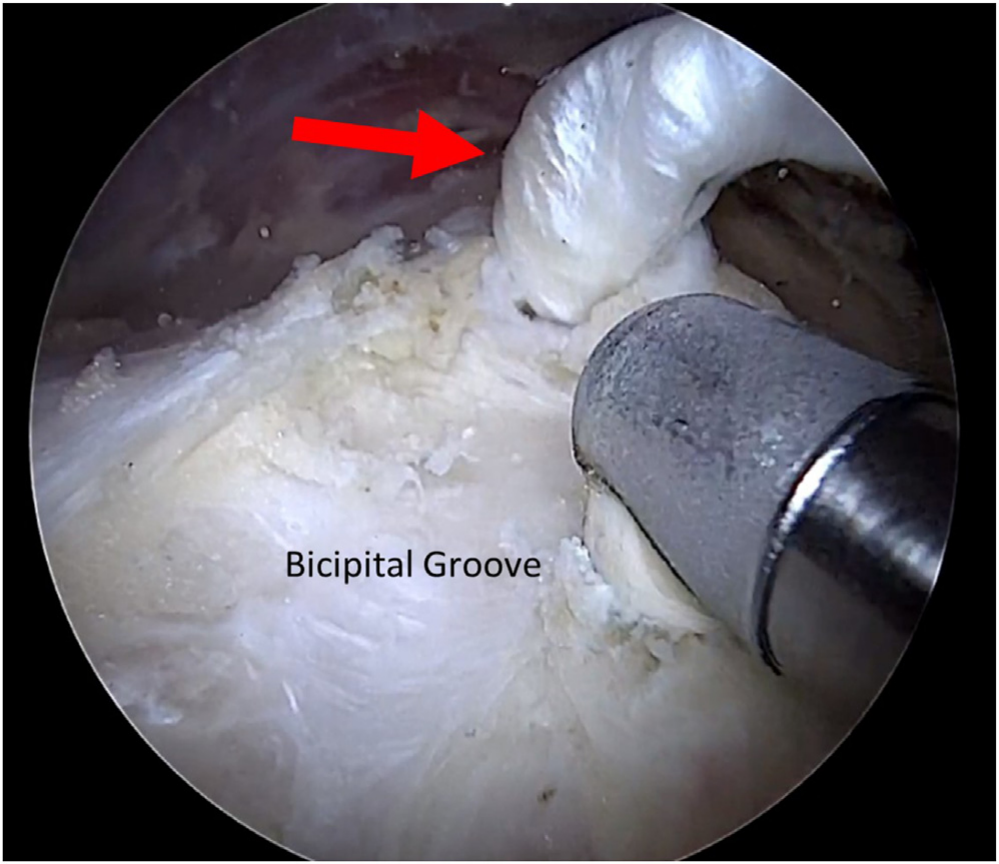
Arthroscopic view from posterior portal with 70° scope in right shoulder. The unsheathed long head of the biceps tendon (arrow) is mobilized into the subacromial space with a grasper. Any remaining tissue in the groove is debrided with a radiofrequency ablation device and/or shaver.

A radiofrequency ablation device, introduced through the lateral portal, is used to incise and open the bicipital sheath, with caution taken to prevent damage to the biceps tendon. The tendon is fully released from the sheath and mobilized from the groove into the subacromial space with a grasper. Any soft tissue within the bicipital groove is debrided with a radiofrequency device and/or shaver (Fig 1).

**Fig 2:**
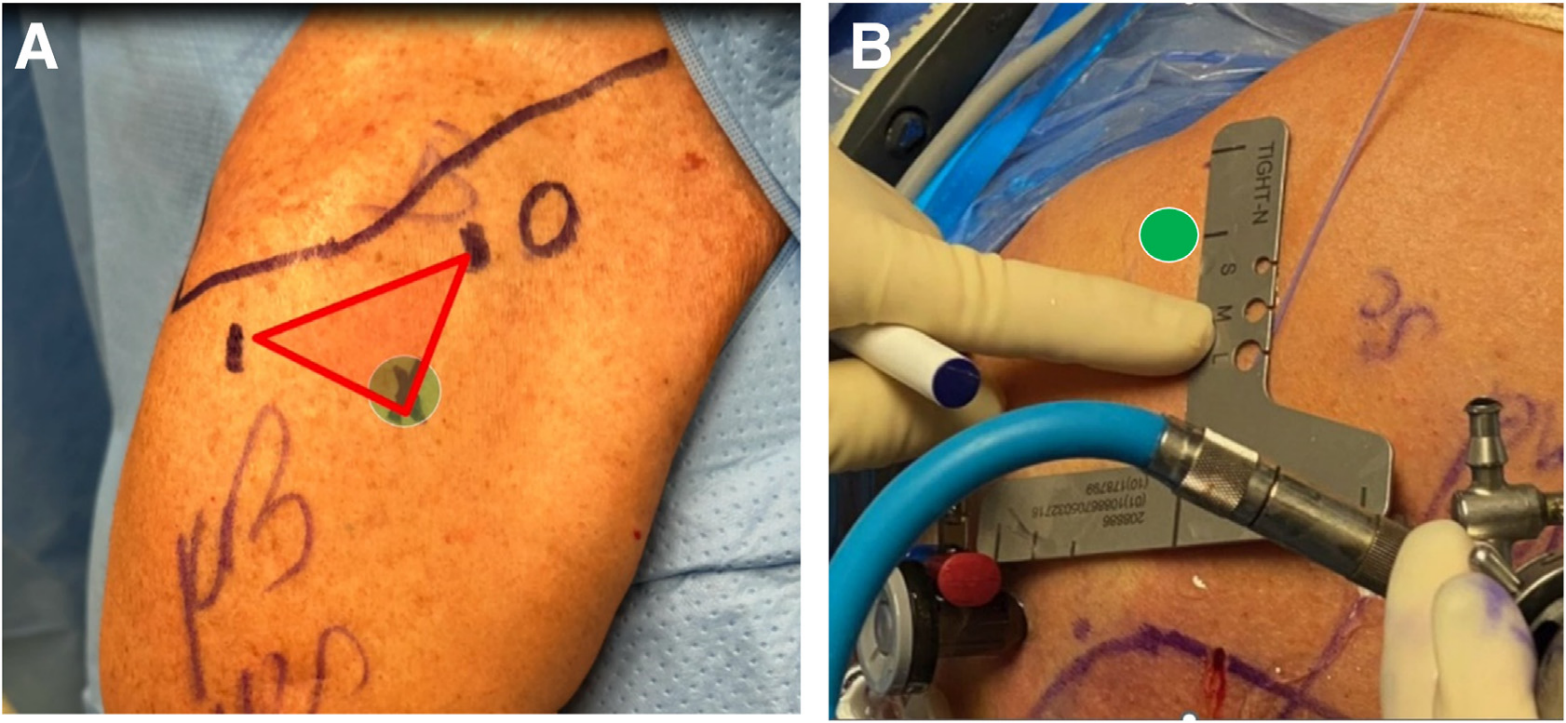
The accessory biceps portal can be located with the triangle method (A) or by use of the sizing guide (B). (A) Exterior view of right anterolateral shoulder in beach-chair position. The triangle method is used to locate the accessory biceps portal by visualizing an isosceles triangle with the anterior portal and lateral portal (red triangle). The tip of the triangle (green circle) indicates the location of the accessory biceps portal. A spinal needle is first inserted and visualized to confirm optimal portal placement. (B) Exterior view of right anterolateral shoulder in lateral decubitus position. The edge of the sizer is aligned with the anterior portal and lateral portal as closely as possible with the same corresponding size lines on either side of the device. The corresponding line length, or position between lines, on the arm of the device indicates the accessory portal location (green circle). In the case shown, the anterior and lateral portals align most closely with the middle lines on the device; therefore, the middle line is marked as the location of the accessory portal. A spinal needle is inserted and visualized to confirm optimal portal placement.

**Fig 3:**
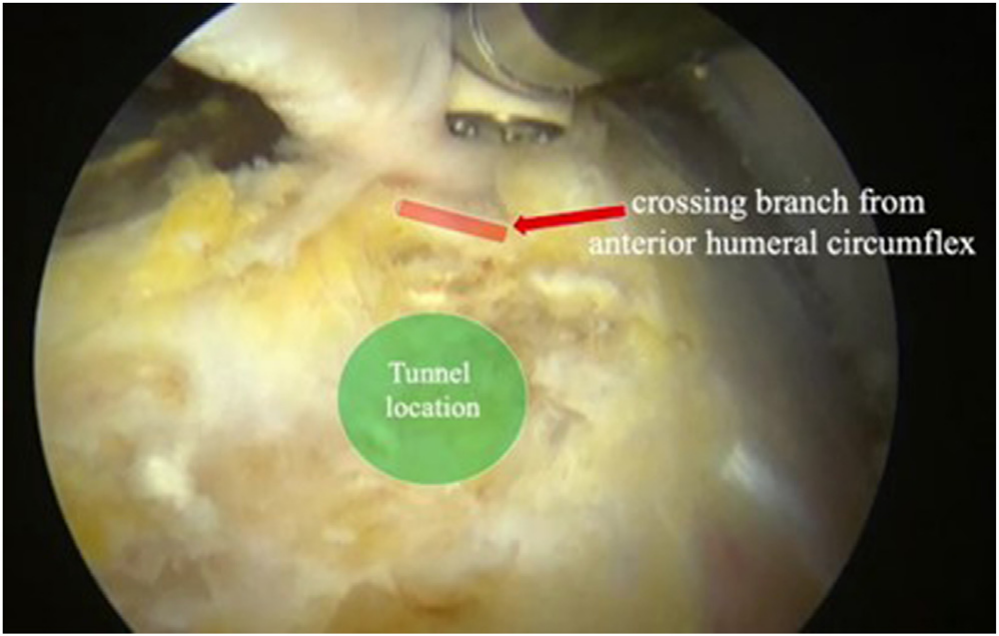
Arthroscopic view from posterior portal with 70° scope in right shoulder. The anterior humeral circumflex vessels (red arrow) traversing the distal bicipital groove can be used as a landmark for the tunnel location. We typically place the bone tunnel approximately 1 cm proximal to these vessels (green circle) in the bicipital groove.

An accessory biceps portal is created at the distal bicipital groove with the aid of a spinal needle to optimize the location. Our typical technique to localize the optimal accessory portal location is the “triangle” method, in which the lateral portal and anterior portal are used to form an isosceles triangle going distally and the third point of the triangle is typically the location of the accessory portal (Fig 2A). Additionally, the TIGHT-N sizing guide, which we have termed the “Getelmeasure,” can be used to locate the accessory portal in a similar fashion (Fig 2B). A spinal needle is used to confirm the location and trajectory of the portal at the distal bicipital groove. There is a consistent branch of the ascending anterior humeral circumflex artery that transverses the distal bicipital groove that can be used as a landmark for placement of the tenodesis tunnel (Fig 3).^16^ This vessel can be cauterized if needed. Although a tenodesis can be performed anywhere in the suprapectoral region with this implant, we prefer to perform the tenodesis distal in the bicipital groove, approximately 1 cm proximal to the anterior humeral circumflex artery.

**Fig 4:**
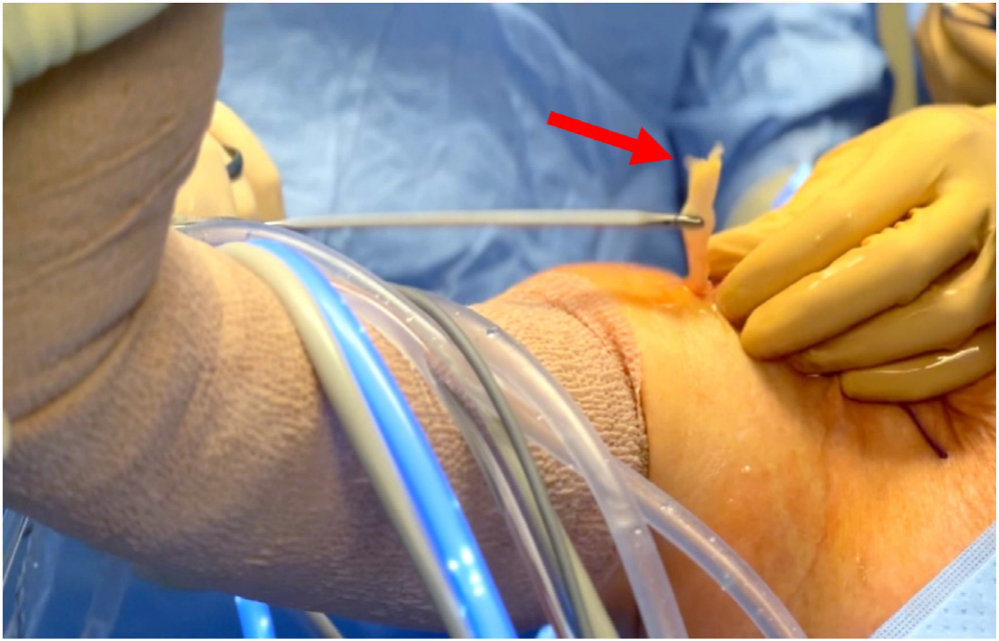
Exterior view of right anterolateral shoulder and extremity. The shoulder and elbow are flexed to aid with exteriorizing a sufficient length of the proximal biceps tendon (arrow) through the accessory biceps portal with a grasper.

**Fig 5:**
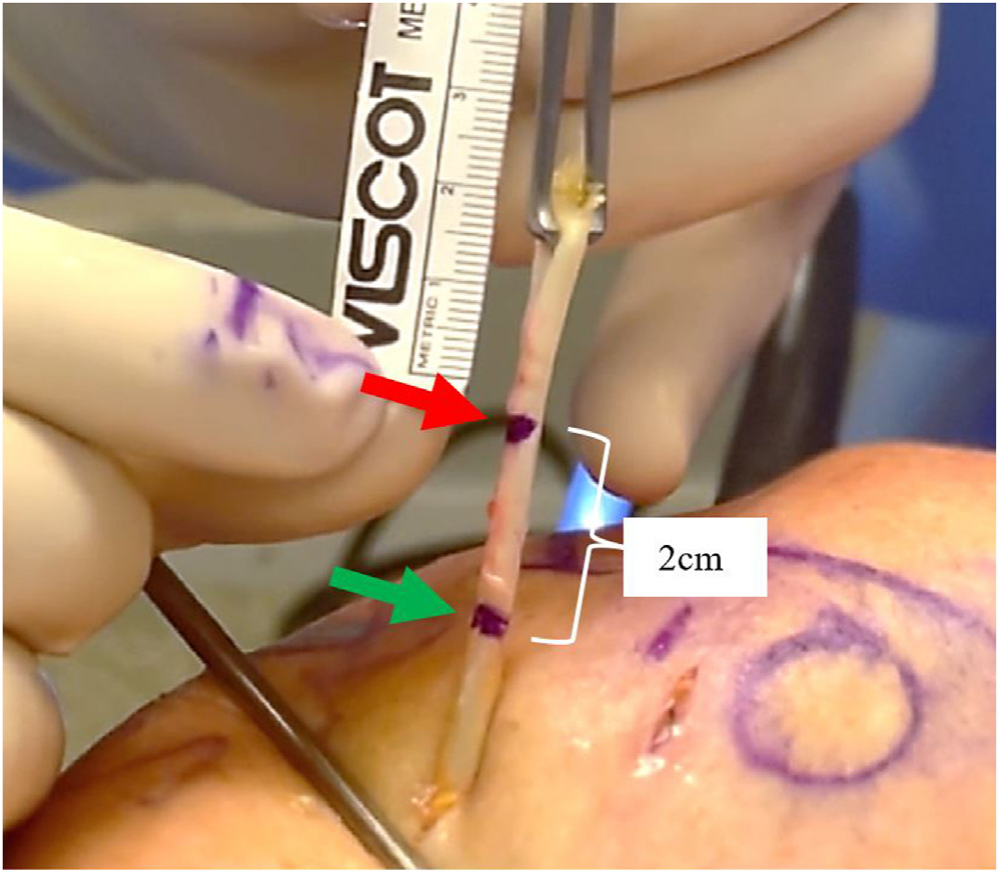
Exterior view of right anterolateral shoulder. The exteriorized biceps tendon (red arrow) is marked in a female patient. This is marked approximately 2 to 2.5 cm from the proximal end in female patients and 3 to 3.5 cm in male patients. A second mark (green arrow) is made 2 cm distal to the initial mark.

**Fig 6:**
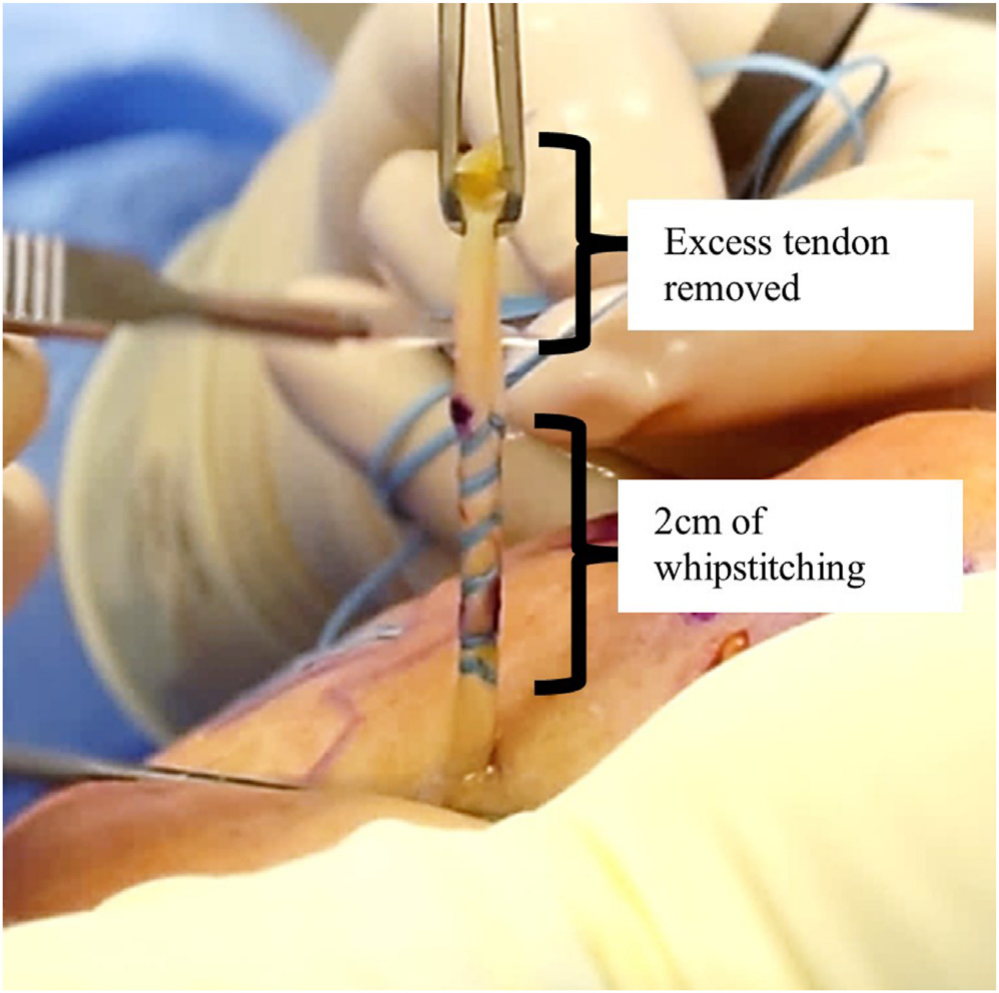
Exterior view of right anterolateral shoulder. The exteriorized tendon is whipstitched from the distal mark to the proximal mark, and the excess proximal tendon is removed with a No. 11 blade.

**Fig 7:**
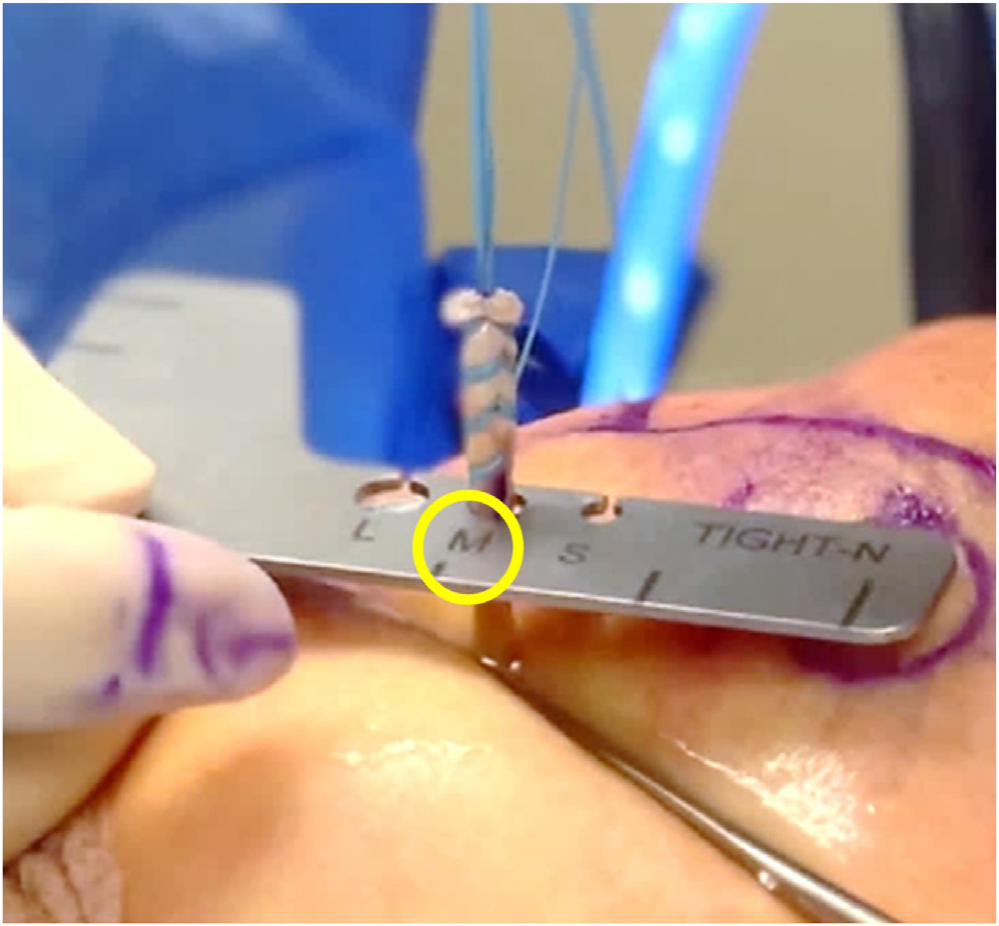
Exterior view of right anterolateral shoulder and TIGHT-N sizing guide. The sutured biceps tendon is sized to find the appropriate anchor and reamer. Options include small (5.5 mm), medium (7.0 mm), and large (8.5 mm). In this case, a medium-sized implant (yellow circle) was selected.

The biceps tendon is retrieved from the accessory biceps portal and delivered out of the skin with an arthroscopic grasper. Shoulder forward flexion and elbow flexion can help exteriorize the necessary biceps tendon length (Fig 4). The proximal aspect of the tendon is held with an Allis clamp. The tendon is marked with a marking pen approximately 2 to 2.5 cm from the proximal aspect of the tendon in female patients and 3 to 3.5 cm in male patients. A second mark is made 2 cm distal to the first (Fig 5). The biceps tendon is sutured with a high-strength, looped 1.3-mm flat braided suture tape (Permaloop; DePuy Synthes) in a whipstitch fashion from the distal mark to the proximal mark, which will allow for appropriate tension in the construct once delivered and secured (Fig 6). The excess proximal biceps tendon is excised. The tendon is appropriately sized using the TIGHT-N sizer to obtain the reamer and implant size (Fig 7). Options include small (5.5 mm), medium (7 mm), and large (8.5 mm).

**Fig 8:**
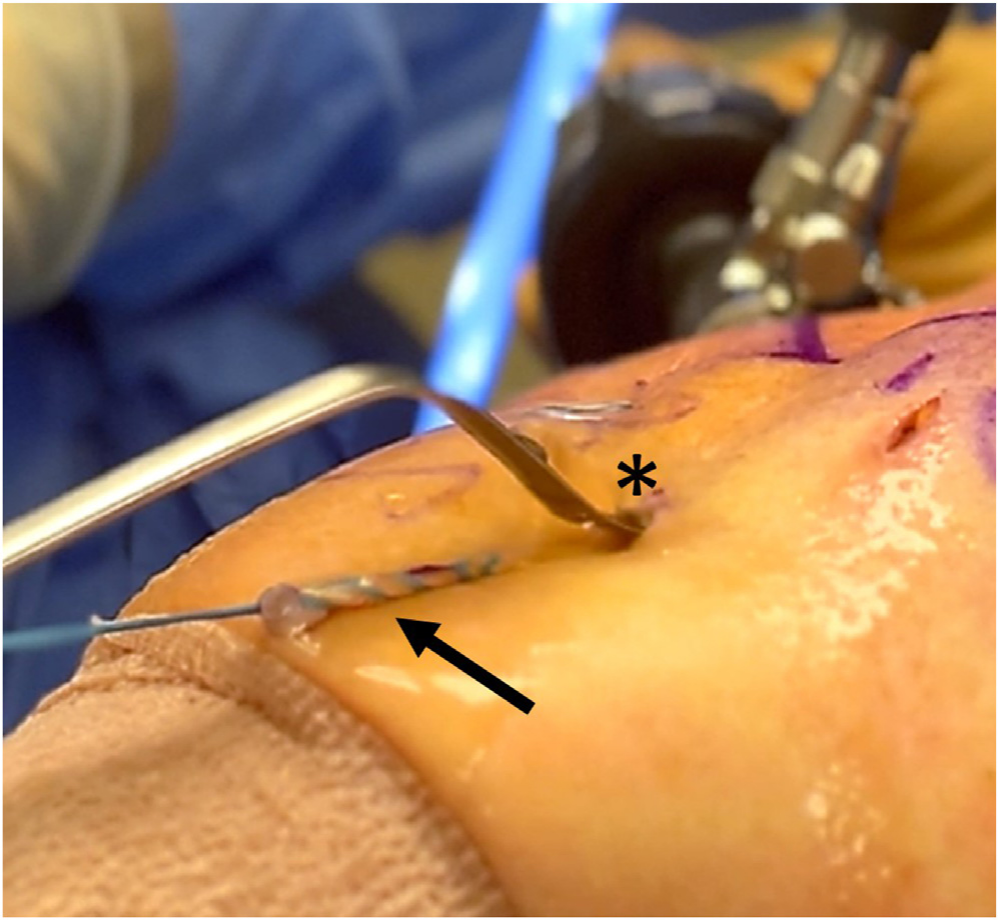
Exterior view of accessory biceps portal in right anterolateral shoulder. A malleable sled retractor is placed in the accessory biceps portal (asterisk) as an alternative to a cannula for reaming. The exteriorized tendon (arrow) is pulled to the side and kept under tension during reaming in cases in which the tendon is sutured and sized first.

There are 2 options for the sequence of steps related to drilling the tunnel. We tend to drill the tunnel first, prior to exteriorizing the tendon, because most of the time, one can select the appropriately sized implant and reamer based on visualizing the tendon relative to arthroscopic instruments. While drilling the tunnel, the tendon is docked medial to the groove in the subacromial space. Once the tunnel is complete, the tendon is exteriorized and prepared. Alternatively, the tunnel is drilled after preparing and measuring the tendon. In the latter case, the prepared tendon is kept under tension and the guide pin and reamer are passed adjacent to the tendon in the same portal. Either way, the guide pin for the cannulated reamer is inserted through the accessory biceps portal and drilled in a unicortical manner within the lower bicipital groove. We find a sled retractor to be a helpful alternative to a cannula (Fig 8). Cannulas are not used in the accessory biceps portal because it would be too challenging to obtain an adequate length of the exteriorized tendon. A cannulated reamer of appropriate size is then used to ream the near cortex to about 23 to 25 mm in depth. This depth allows for the 18-mm TIGHT-N implant with the 2-mm knot stack to be buried in a subcortical manner. The reamer and guidewire are removed. A shaver is introduced to remove debris, and a radiofrequency device can be used to circumferentially remove soft tissue from the drill hole.

**Fig 9:**
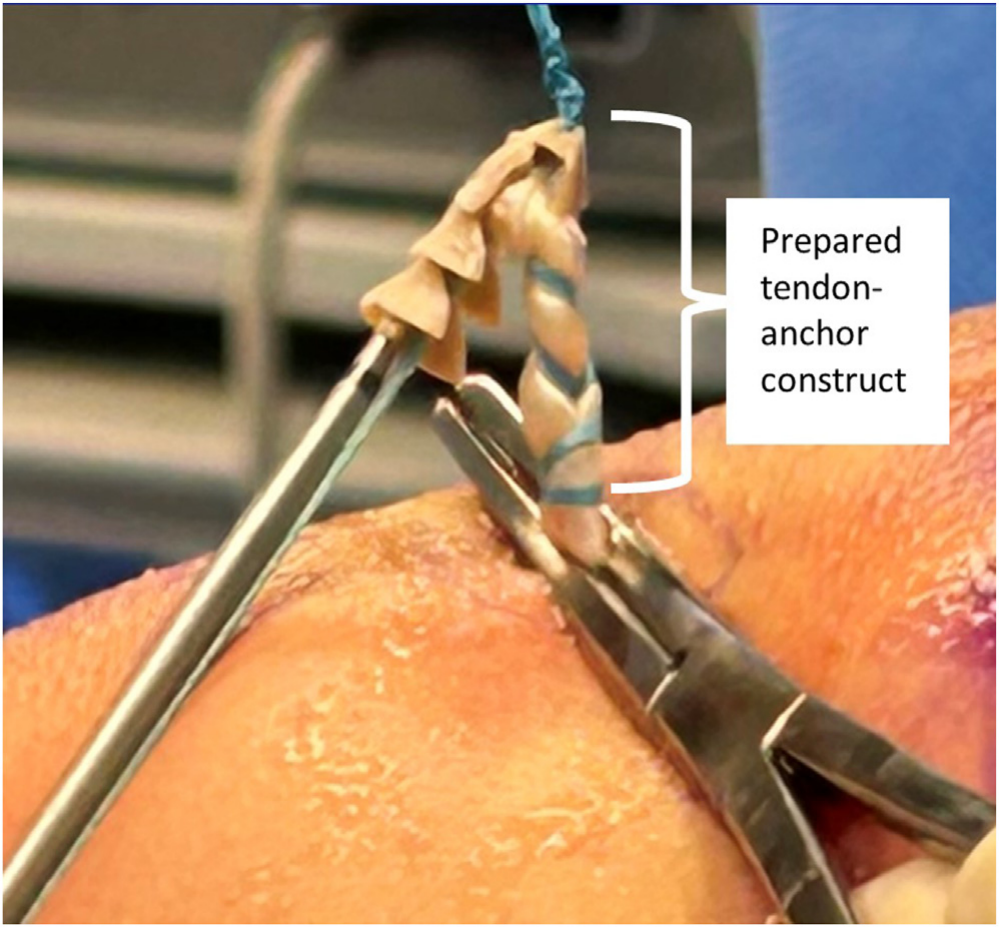
Exterior view of anterolateral shoulder with prepared implant-tendon construct. The distal portion of the tendon is clamped at the skin to allow for easier management and docking of the tendon to the TIGHT-N device.

**Fig 10:**
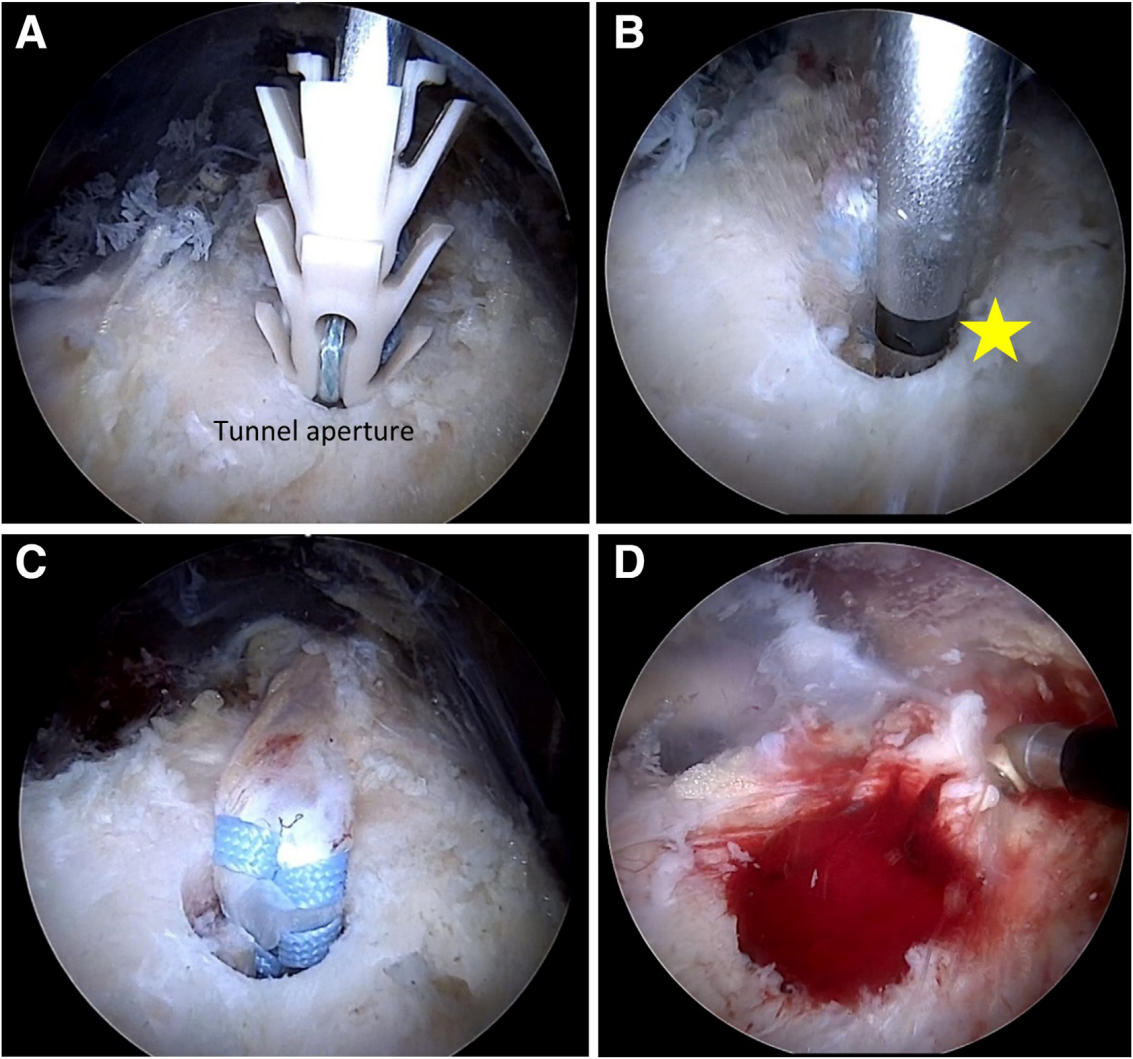
Arthroscopic images obtained with 70° scope from posterior portal in right shoulder. (A) Proximal view of loaded TIGHT-N anchor prior to insertion into bone tunnel in the distal bicipital groove. The handle of the inserter is tapped very lightly with a mallet until the implant-tendon construct is inserted to the level of the black line (22-mm mark). (B) View of black line on inserter that is flush with cortical bone surface (yellow star), indicating optimal implant depth. (C) View of completed biceps tenodesis using TIGHT-N implant. (D) View of tunnel aperture filled with marrow elements and blood clot from the tunnel minutes after the tenodesis is completed.

The biceps tendon is then docked onto the TIGHT-N implant. The distal portion of the exteriorized tendon can be clamped at the skin to allow for easier management and securing of the tendon to the TIGHT-N device (Fig 9). Each whipstitch strand is inserted through the eyelets at the leading nose of the implant to load the biceps onto the implant. The tendon is pulled up to the leading end of the implant and placed under the nose clip to enhance delivery of the tendon into the tunnel. A minimum of 5 secure knots with alternating half-hitches are then tied to secure the tendon to the implant. The implant with the docked biceps tendon is placed through the accessory portal to the aperture of the tunnel. A malleable sled retractor can be introduced through the accessory biceps portal to assist in the delivery of the implant through the deltoid. Very light tapping with a mallet onto the insertion handle is performed to insert the implant-tendon construct into the tunnel to the black line (22-mm mark). The sutures that secure the insertion handle to the implant are unwound and removed so that the insertion handle can be removed (Fig 10). Appropriate tension is then confirmed with a probe.

Most patients use a sling for 6 weeks while avoiding active elbow flexion and forearm supination. However, we have treated a cohort of isolated BT patients who have weaned off sling use within 7 to 10 days of surgery without failures thus far. After 6 weeks, patients can progress to active-assisted range of motion with subsequent advancements to active range of motion as tolerated. Initial strengthening can begin at approximately 8 weeks, with more advanced strengthening at 10 to 12 weeks.

## Discussion

A multitude of tenodesis techniques for the treatment of LHBT pathology have been developed over the years. Often, when there are so many options, it means that everything works well or that an optimal solution has not yet been found. Although the approach using an IS in a bone tunnel has shown biomechanical equivalence and advantages compared with other tenodesis methods, it has significant limitations. There is a risk of the screw lacerating the biceps tendon on insertion at the cortical interface, which can lead to relatively high rates of tenodesis failure.^17^ Although IS fixation was initially very popular for inlay tenodesis, many surgeons have shifted toward onlay tenodesis with suture anchors or buttons to decrease delayed postoperative failures seen with IS techniques.^18^, ^19^, ^20^ Park et al.^10^ reported a 21% failure rate in patients treated with IS fixation versus a 6% rate in onlay tenodesis patients. Similarly, Haidamous et al.^19^ found postoperative Popeye deformities in 27% of patients with inlay fixation compared 9.4% of patients with onlay fixation. Finally, Jackson et al.^4^ reported Popeye deformities in 11.3% of the inlay patients and 7.8% of the onlay patients in their meta-analysis comparing the two techniques.

Conversely, recent studies have reported that onlay fixation may have downsides related to postoperative tendon healing and elongation. Forsythe et al.^13^ found 2- to 3-fold greater tendon migration at 3-month follow-up in patients receiving single-suture suture anchor onlay fixation compared with IS inlay patients, regardless of fixation location. In fact, 28.1% of patients in the open subpectoral onlay group (1 suture anchor) and 15.4% of patients in the suprapectoral onlay group (2 suture anchors) experienced tendon migration greater than 3 cm and were considered to be radiographic failures, indicating compromised tendon-to-bone healing. Similarly, Trefzer et al.^14^ observed an average of 3 to 3.8 cm of distalization of the myotendinous junction of the LHBT with subpectoral button onlay fixation at minimum 2-year follow-up. It has been recently reported by Forsythe et al. that a 1-cm increase in post-tenodesis biceps tendon migration (following suprapectoral or subpectoral tenodesis) was associated with lower patient-reported outcome scores. ^21^ It is evident that onlay techniques can lack healing predictability and that where the tendon is fixed at time zero is often not the final resting position of the tendon and that tendon elongation or migration can adversely effect outcomes.^13^^,^^14^^,^^21^ Thus, we believe these published rates of onlay failures are still too high and can be improved, which was the motivation behind the implant design described in this article.

Tunnels or even small anchors in the subpectoral region can increase the risk of postoperative fracture, but this has not been the case with tunnels in the suprapectoral location.^12^^,^^20^ The device used in our technique, which requires drilling a unicortical tunnel, is recommended for use in the suprapectoral zone of the proximal humerus but not the subpectoral region. This implant comes in 3 sizes: 5.5, 7, and 8.5 mm. The tunnel diameter is the same as the implant size. In our experience, the 7-mm implant is used 65% to 70% of the time and the 5.5-mm implant is used in the remainder. Although subpectoral fractures have caused many surgeons to avoid tunnels in this diaphyseal location, to our knowledge there have been no reported postoperative fractures after suprapectoral tenodesis with tunnels. In fact, in a recent cadaveric model in which suprapectoral tunnels were created and the proximal humerus was loaded, fractures typically occurred in the subpectoral location and not through the suprapectoral tunnels.^12^ Despite the concern of creating stress risers with tunnels, this has not been an issue with suprapectoral tunnels.

The implant reported in this article attempts to address the pitfalls of current inlay and onlay constructs. The half-sheath design protects against iatrogenic damage and delivers the tendon into a suprapectoral tunnel. The healing environment with this technique allows access to the intramedullary marrow elements and facilitates tendon healing at the cortical aperture of the tunnel. The PEEK flanges of the device compress when deployed and expand once seated in a subcortical manner, providing strong fixation with low variability.^15^ Although this technique can be performed as an open procedure, the implant and technique were designed for straightforward arthroscopic suprapectoral delivery. Even novice surgeons should be able to obtain proficiency with this technique quite quickly. The ideal BT procedure must be safe, must be easily reproducible, and must yield strong results while minimizing failures. The TIGHT-N Anchor was designed to surmount the shortcomings of current techniques and has the potential to benefit many patients undergoing BT by reducing delayed failures and providing strong fixation strength.

## Disclosures

The authors declare the following financial interests/personal relationships which may be considered as potential competing interests: K.F.B. reports board membership with the Arthroscopy Association of North America and LifeNet Health; owns equity or stocks in COVR Medical and Zimmer Biomet; reports a consulting or advisory relationship with DePuy Synthes, Embody, Regenity Biosciences, and Zimmer Biomet; receives speaking and lecture fees from DePuy Synthes and Zimmer Biomet; receives funding grants from LifeNet Health and Wolters Kluwer Health; has patents with royalties paid to DePuy Synthes and Zimmer Biomet; is one of the developers of the TIGHT-N Anchor; and receives intellectual property royalties from DePuy Synthes. All other authors (M.G.S., K.S.W., T.E.H.) declare that they have no known competing financial interests or personal relationships that could have appeared to influence the work reported in this paper.

## Supplementary Data

Video 1 Inlay arthroscopic suprapectoral biceps tenodesis technique using tendon-docking anchor (TIGHT-N Anchor). The long head of the biceps tendon is released from its attachment on the glenoid, and a thorough bursectomy is completed. Viewing from the posterior portal with a 70° scope angled anterior and distal in the subacromial space, the biceps tendon is located in the bicipital groove. The groove is carefully opened and decompressed with a radiofrequency device, and the tendon is delivered medially into the subacromial space. The appropriately sized implant and reamer are selected, and the bone tunnel is reamed from the accessory biceps portal to approximately 22 to 25 mm in depth. The senior author (K.F.B.) prefers to place the tunnel more distal in the groove, approximately 1 cm proximal to the crossing branch of the anterior humeral circumflex vessel. The accessory biceps portal is next located using the triangle method and first visualized with a spinal needle for optimal placement. The tendon is retrieved with a grasper and delivered through the accessory biceps portal. The tendon is marked 2 to 2.5 cm along the tendon in female patients and 3 to 3.5 cm in male patients. A second mark is made 2 cm to the first mark. The tendon is then sutured in whipstitch fashion from the distal mark to the proximal mark, and the excess proximal tendon is removed. The prepared tendon is docked to the TIGHT-N anchor and secured with a minimum of 5 secure knots with alternating half-hitches. The loaded implant is delivered through the biceps portal to the aperture of the tunnel and gently tapped into place to the black line, indicating appropriate depth. The completed tenodesis is then checked with a probe.
